# Multiple social roles in early adulthood and later mental health in different labour market contexts

**DOI:** 10.1016/j.alcr.2021.100432

**Published:** 2021-06-25

**Authors:** Miriam Engels, Morten Wahrendorf, Nico Dragano, Anne McMunn, Christian Deindl

**Affiliations:** a Institute of Medical Sociology, Medical Faculty, Heinrich-Heine-University Düsseldorf, Moorenstr. 5, 40225 Düsseldorf, Germany; b Research Department of Epidemiology & Public Health, University College London, 1-19 Torrington Place, London WC1E 6BT, UK; c Faculty of Social Sciences, TU Dortmund University, Emil-Figge-Straβe 50, 44227 Dortmund, Germany

**Keywords:** Gender, Depression, Life course, Labour market, Work-family, Multiple roles

## Abstract

Work and family roles entail divergent responsibilities, which can be a source of conflict especially in young adulthood – the so-called “rush-hour” of life. Combining these multiple social roles can result in an accumulation of stress but also be a valuable resource for mental health. The aim of this study is to investigate combined employment, parenthood and partnership trajectories of men and women during early adulthood, and to analyse the relationship of these multiple roles with depressive symptoms at older age.

We used harmonised data from the Survey of Health, Ageing and Retirement in Europe (SHARE) and the English Longitudinal Study of Ageing (ELSA) with retrospective information on employment, partnerships and parenthood histories between age 25 and 40 for 18,816 men and 24,686 women (n = 43,502). We applied sequence analysis and clustering to group trajectories into four clusters for women and three clusters for men. We then used multilevel models to analyse the links between combined employment and family roles and later mental health in different historical labour market contexts (indicated by female employment rates).

Women and men who did not combine work and family roles between age 25 and 40 report higher levels of depression than those who combined work and family. Results differ by gender and labour market context, with stronger differences between women in countries with higher female employment rates.

Overall, combining multiple roles in early adulthood is associated with decreased rather than increased risk for depressive symptoms in older Europeans.

## 1. Introduction

In light of ageing populations in most parts of the world, maintaining mental functioning at older age is one of the main public health challenges for societies. A major influence on mental health at older age is the accumulation of stressors and resources within different social roles over the life course (Dannefer, 2003; Deindl, 2013; Ferraro & Schafer, 2017; Kuh, Ben-Shlomo, Lynch, Hallqvist, & Power, 2003). The most salient social roles over the life course are employment, partnership and parenthood: While paid work can serve as an important source for income and wealth, social support and identity, stressful working conditions, episodes of unemployment or job insecurity can also have a long lasting, negative impact on one’s mental health (Tausig, 2013; Thoits, 2011). Similarly, living with a partner and starting a family are associated with better social support and increased satisfaction but also create increased demands on time and energy that have been shown to affect mental health and well-being later in life (Evenson & Simon, 2005; Nelson, Kushlev, English, Dunn, & Lyubomirsky, 2013).

For most adults, employment, parenthood and partnership are heavily interconnected roles and changes in one social role heavily affect the other social roles. Therefore, it is important to investigate the interplay of different social roles and the association between certain combinations of work and family situation and to look at the combined effect of all three on mental health in later life. The relationship between work and family life and the potential stress resulting from combining these roles can differ not only between men and women but also between countries. In conservative countries, where the male breadwinner model is dominant, combining family with work might have a different effect on mental health than in Northern countries or Eastern European countries with their longer history of female employment (e.g. Esping-Andersen, 1999). Surprisingly, we know little about the impact of combining social roles in different countries and their relationship with mental health later in life. The aim of this study is therefore, to investigate the relationship between multiple roles and mental health from a life course perspective, and to compare these associations for older men and women across different labour market contexts.

### 1.1. Work-family combinations and mental health

There are important differences between men and women in the way that work and family roles are combined. While men, in general, mostly continue to work full-time throughout adulthood regardless of their family situation, the work-family constellations of women during adulthood are more diverse. In older cohorts, married women with children typically engaged mostly in unpaid domestic work, at least temporarily, while their husbands continued to be employed (Worts, Corna, Sacker, McMunn, & McDonough, 2016). When the Baby Boom generation (born after 1945) came of age, gender role expectations started to change rapidly and the women’s movement had led to an increase in mothers who continued their employment on top of their family roles (Carr, 2002). This has led to an increase of studies on the psychological consequences of these changes in work-family role constellations.

Combining work and family can be either stressful of fulfilling and it therefore lead to the ideas of role strain (or role stress) on the one hand, and role enhancement (also role expansion) on the other (Rozario, Morrow-Howell, & Hinterlong, 2004). Role strain theories suggest that multiple roles (such as employee, father/mother and husband/wife) form competing demands on limited time and energy of the individual, with consequent increased risks for conflict and stress (Goode, 1960; Marks, 1977). On the other hand, role enhancement theories (Sieber, 1974) argue that multiple roles can also act as resources to counterbalance higher demands. Being employed gives access to financial resources (i.e. higher income, better insurance or pensions) while having a family gives access to social resources (i.e. bigger social networks, more social support and equality). Especially for women, combining family with paid work, if voluntary, can increase agency and lead to better well-being (McMunn, Bartley, & Diana, 2006). But it can also increase stress, especially during early adulthood, the so called “rush-hour” of life, when starting a career and family life come together and the potential for conflicts between divergent responsibilities is at a peak (Lippert & Damaske, 2018; Zannella, Hammer, Prskawetz, & Sambt, 2018) or when partnerships dissolve and work and parenting responsibilities have to be carried alone.

Over the last decade researchers have started to investigate the long-term mental health impacts of combined work and family roles (Moen, 2011; O’Flaherty, Baxter, Haynes, & Turrell, 2016). Increased availability of longitudinal data (either collected prospectively or retrospectively) has led to a growing number of studies that highlight the relevance of conditions in early life (e.g. childhood circumstances and educational attainment) for work-family trajectories in adulthood (Damaske & Frech, 2016; Worts, Sacker, McMunn, & McDonough, 2013). More recently, studies also investigated the relationship between work-family trajectories and later mental health (Arpino, Gumà, & Julià, 2018; McMunn et al., 2016). One stream of life course theory suggests that social stressors can accumulate over the life span and make individuals more vulnerable to depression at older age (Dannefer, 2003; Juster, McEwen, & Lupien, 2010). At the same time, financial and psychosocial resources (e.g. social support or agency) can also accumulate and buffer against depression at older age (Becker, Kirchmaier, & Trautmann, 2019; Butterworth, Rodgers, & Windsor, 2009). Studies have also shown that trajectories of continuous paid work in adulthood are generally related to better socio-economic conditions and mental health later in life (Freeh & Damaske, 2012; Wahrendorf, Blane, Bartley, Dragano, & Siegrist, 2013). However, studies on the effects of combining paid work with parenthood differ in their results. Some studies indicate that combining paid work with family roles is beneficial for mental well-being at older age (Lacey et al., 2016; McMunn, Bartley, Hardy, & Diana, 2006; Stone, Evandrou, Falkingham, & Vlachantoni, 2015). Others show no relationship with depressive symptoms or even a negative association with mental well-being when combining full-time work with childcare responsibilities (Benson et al., 2017; Di Gessa, Corna, Price, & Glaser, 2020; Engels et al., 2019). These studies suggest that role conflicts do not just depend on the number of social roles but on the specific combination of roles. Some constellations are particularly vulnerable for depression later in life, including combining work with early or single parenthood (McMunn, Bartley, Hardy et al., 2006). Recent studies underline that the specific combinations of multiple social roles are shaped by gender-specific role expectations and country-specific policies and social norms (Zagel & Van Winkle, 2020).

### 1.2. Cross-country differences

The differences between countries, when it comes to the mental health effects of combining work and family roles (Artazcoz et al., 2014), are in line with the “principle of time and place” of life course theory (Elder, Johnson, & Crosnoe, 2003), which emphasises that historical and socio-cultural circumstances restrict the choices of individuals at any given time. Cultural norms and social policies can shape what is expected of men and women in early adulthood, and how much support individuals receive when it comes to combining paid employment and family responsibilities, such as unpaid childcare and domestic work (Hagqvist, Toivanen, & Bernhard-Oettel, 2018; Leitner, 2004). For example, familialistic policies strengthen the caring function of the family by providing generous paid parental leave, while de-familialistic policies are characterised by the provision of extensive public childcare services (Saraceno & Keck, 2011). A welfare context that largely institutionalises childcare increases the likelihood that women enter the labour market in early adulthood (Cukrowska-Torzewska, 2017). This can, for example, be observed in former communist countries in Eastern Europe or Nordic countries, where employment rates among women were on average higher than in Western and Southern European countries which support more traditional divisions of work and family roles between men and women (Grunow, Begall, & Buchler, 2018). Therefore, the labour market situation in a given time period, especially female employment rate, reflects countries’ cultural norms and policies at the time, and may affect the relationship between multiple roles and mental health.

### 1.3. Aims of the current study

The aim of this study is to bring these aspects together and to investigate the relationship between multiple roles and mental health from a gendered life course perspective across different labour market contexts. For this purpose, we analyse life history data of older men and women from 22 European countries to find the most common combinations of employment, parenthood and partnership roles across early to mid-adulthood for each gender and relate them to mental health at older age. Additionally, we set out to test whether this relationship differs by labour market context at the time.

## 2. Methods

### 2.1. Data

We used harmonised life history data of the Survey of Health, Ageing and Retirement in Europe (SHARE) (Börsch-Supan, Brandt, Hunkler et al., 2013; Börsch-Supan, Brandt, & Schröder, 2013; Börsch-Supan et al., 2019) and the English Longitudinal Study of Ageing (ELSA) (Steptoe, Breeze, Banks, & Nazroo., 2013) provided by the Gateway to Global Ageing (Wahrendorf, Deindl, Beaumaster, Phillips, & Jinkook, 2019; Wahrendorf, Deindl, Beaumaster, Phillips, & Jinkook, 2019). Both surveys follow a similar design and have a representative sample of individuals aged 50 and above as well as their (possibly younger) partners, with interviews in two-year intervals. ELSA started in 2002, SHARE in 2004. SHARE started in eleven countries, with additional countries joining and some countries leaving the study over time. Combined over all waves, SHARE offers information on 29 countries, of which 22 countries measure depression at older age. Apart from regular interviews on current circumstances ELSA and SHARE conducted life history interviews in their third wave in which respondents provided detailed retrospective information about their whole life course. SHARE, additionally, included a life history questionnaire again in their current wave (wave 7) including all respondents who did not take part in the previous life history interview. These life history datasets include detailed information about five key domains: children, partnership, housing, work, and health. The Gateway to Global Aging Data Platform provides harmonised data from SHARE and ELSA for each of these five domains in a state sequence format with annual information from age 15 till age 80.

### 2.2. Sample

We used a pooled sample of waves 1–7 of SHARE and waves 1 and 3 of ELSA. For each respondent we used information from the first interview he/she conducted and combined those with retrospective information from their life history interviews. The pooled data of SHARE (wave 1–7) and ELSA (wave 1 and 3) has 92,534 valid cases from 22 European countries: Austria, Belgium, Croatia, Czech Republic, Denmark, England, Estonia, France, Germany (former West Germany), GDR (former East Germany), Greece, Hungary, Ireland, Italy, Luxembourg, Poland, Portugal, Slovenia, Spain, Sweden, Switzerland and the Netherlands.

Life histories in SHARE and ELSA cover a period from 1915 until today. Especially in the first part of the last century, the life courses of most Europeans were interrupted by two world wars. In order to allow for uninterrupted work histories, we restrict our analysis to those born after World War II (1945). Of the 52,667 respondents born after 1945, we distinguish between two age groups (‘cohorts’): respondents born between 1945 and 1954 and respondents born afterwards. 9,165 had missing states for their employment, fertility or partnership history between the age 25 and 40, leaving us with 43,502 respondents with complete states for our sequence analysis.

Two factors determine the age limits of our sequence analysis. The first factor is the time frame where life courses are most interesting - formative years for work and family. The “rush-hour" of life, described in the introduction, lies between the age of 25 and 40, when people have finished their education and begin to establish their career as well as their family life. The second factor is the availability of a macro indicator to capture macro level influences on the relation between work-family sequences and depression. The earliest available macro indicators for labour market context start in 1970 and therefore align with the age limit of 25 of our oldest cohort born 1945. Our sample includes 24,686 female and 18,816 male respondents. Additional missing values for depression and other variables in our main model reduced the sample for the multi-level analyses to 22,105 women and 16,379 men.

### 2.3. Dependent and independent variables

#### Depression:

Our main dependent variable is current mental health measured through pooled depressive symptoms. SHARE uses Euro-D as measurement for depression (see Prince et al., 1999 for details on Euro-D). Euro-D includes 12 items describing common depressive symptoms, asking if these symptoms occurred over the past week. Where participants answered all of the items, an additive sum score is available for each wave that the subjects participated in, ranging from 0 to 12, with higher values indicating more depressive symptoms. ELSA uses CES-D to measure depression (Andresen, Malmgren, Carter, & Patrick., 1994; Radloff, 1977). Here people answered eight questions about the occurrence of depressive symptoms in the past week with a sum score ranging from 0 to 8. Despite differences in the range of their sum scores, both scales have shown to have similar associations with risk factors for depression (Courtin, Knapp, Grundy, & Avendano-Pabon, 2015). For our analyses, we use z-standardised scores of Euro-D and CES-D to make SHARE and ELSA comparable.

*Work-family histories* of the respondents between age 25 and 40 are our main independent variable. Employment, parenthood and partnership histories are derived from retrospective life history interviews of SHARE and ELSA, using the so-called life-grid approach. The data derived from these interviews include dates of all important life events, such as birth or adoption of any children, entering and leaving partnerships, and detailed information on each job, including starting and ending dates, as well as information on episodes before the first job, between jobs and after the last job. From this information, the harmonised life course dataset (Wahrendorf et al., 2019a) reconstructs an individual state for each different role: a) employment - having a job (yes or no), b) partnership - living together with a partner (yes or no), and c) parenthood - having a child under 18 (yes or no). These states allow for eight combinations for each year: 1. Work, no children, no partner, 2. Work, children, no partner, 3. Work, no children, partner, 4. Work, children, partner, 5. No work, no children, no partner, 6. No work, children, no partner, 7. No work, no children, partner, 8. No work, children, partner. These states formed the basis for the sequence analysis described below.

*Labour market context* was measured by average female employment rate of each country around the age of labour market entry taken from the Yearbooks of the International Labour Organziation (ILO) (International Labour Organization, 1944, 1954, 1961, 1969, 1979, 1990) and the Labour Market Survey (International Labour Organization, 2019). For this, we take official statistics on employment rates from the 1970s and 1980s respectively. These historical employment rates are not available for every year, which is why we calculate the average employment rate of the decade in which the respondents were in their twenties (see Table A1 in Appendix).

#### Additional Variables:

To address potential health selection effects, childhood health was considered in the models and assessed with a binary variable indicating if the individual was ever hospitalised for at least one month (from birth up to the age of 15 years). The following variables are added to control for socio-economic circumstances over the life course: Childhood circumstances are indicated by living conditions at the time respondents were at the age of 10. Living conditions were operationalised by overcrowding, i.e. the number of people per room in the household. Education was used as an indicator for socio-economic conditions just before entering early adulthood and as a determinant of later life social mobility. For education (as an independent variable) we distinguish between low, medium and high education, where “high” means tertiary education (ISCED = 5-6), “medium” means secondary education (ISCED = 3-4), and” low” means primary education (ISCED = 0-2) (Unesco, 1997). Additionally, we control for income and wealth as indicators for current socio-economic conditions at older age. SHARE provides information of income and wealth as household characteristic. In contrast to this, ELSA measures both income and wealth on the benefit unit level. We therefore use the harmonised Gateway version, which measures both quantities on the benefit unit level. Similar to the new OECD-equivalent scale, we weighted income in both studies by household size, where the first member of the household has a weight of 1 and each additional member is given weight of 0.5. To make comparison between countries easier we constructed 10 percentiles for income and wealth for each country.

### 2.4. Analytic strategy

We applied sequence analysis to group similar work-family histories into clusters. These clusters were then used in a multilevel model to assess their relation with depression. We expected women and men to differ substantially in their life courses, therefore cluster analysis was performed separately for men and women.

First, we calculated the average years spent in each of the eight work-family states and the mean number of spells. This allowed us to see how many years individuals spent combining multiple roles and how many changes in roles they went through between age 25 and 40. We then used Optimal Matching (OM) to quantify the distances between each pairs of work-family sequences with substitution costs set to 1 and indel costs set to 0.5 (Abbott & Tsay, 2000), assuming the same valence for all changes in work-family constellations. We used the resulting distance matrix to group sequences into clusters using cluster analysis (“Ward’s linkage”). To determine the appropriate number of clusters we compared solutions between two and five clusters based on quality measures, cluster sizes and content validity. We preferred a four-cluster solution for women and a three-cluster solution for men, which are supported by PBC and ASW (see Table A2 in Appendix) and had a reasonable solution. We also tried alternative cluster formations, where some of the less frequent work-family states where grouped together (e. g. for single parents “work, kids, no partner” and “no work, kids, no partner”) before comparing the sequences. These resulted in similar cluster solutions with comparable quality measures but smaller cluster sizes. We therefore decided to continue the analysis with the cluster solution based on eight states. We used the TraMineR, WeightedCluster, and Cluster package in R for the sequence analysis (Gabadinho, Ritschard, Müller, & Studer, 2011; Studer, 2013).

We investigated the links between work-family clusters and mental health later in life and whether this link is moderated by historical labour market contexts. We used multi-level linear regression models. Our dependent variable was pooled depressive symptoms. To analyse the role of labour market context, we included female employment rate as a level 2 variable in our second model and we also calculated cross-level interactions between work-family cluster and female employment rate in a third model. All models are calculated separately for men and women and adjusted for age (centered) and age squared.

Because the distribution of depressive symptoms was skewed to the right, we also calculated models based on the transformed scale (natural logarithm), where distributions were normal (Wooldridge, 2009). The analyses yielded similar results. For ease of interpretation the Results section presents regression estimates based on non-transformed scores for number of depressive symptoms. All calculations of multivariate models and margin plots are based on Stata 15.

## 3. Results

### 3.1. Sample description

Table 1 provides a description of the sample and depicts the differences between men and women. In our sample, compared to men, women are a little bit younger, more often have low education and report more depressive symptoms.

Table 1 also shows how many years between age 25 and 40 men and women spent on average in each work-family constellation (see *Cumulative durations*). It reveals some important gender differences in dominant life course social roles. Men spent on average more years combining paid work with family than women, while women spent more years without paid work, especially mothers. Interestingly, both men and women spent most years combining all three roles (employment, parenthood and partnership) between the ages 25 and 40.

### 3.2. Multiple roles across the life course

**Fig. 1.**
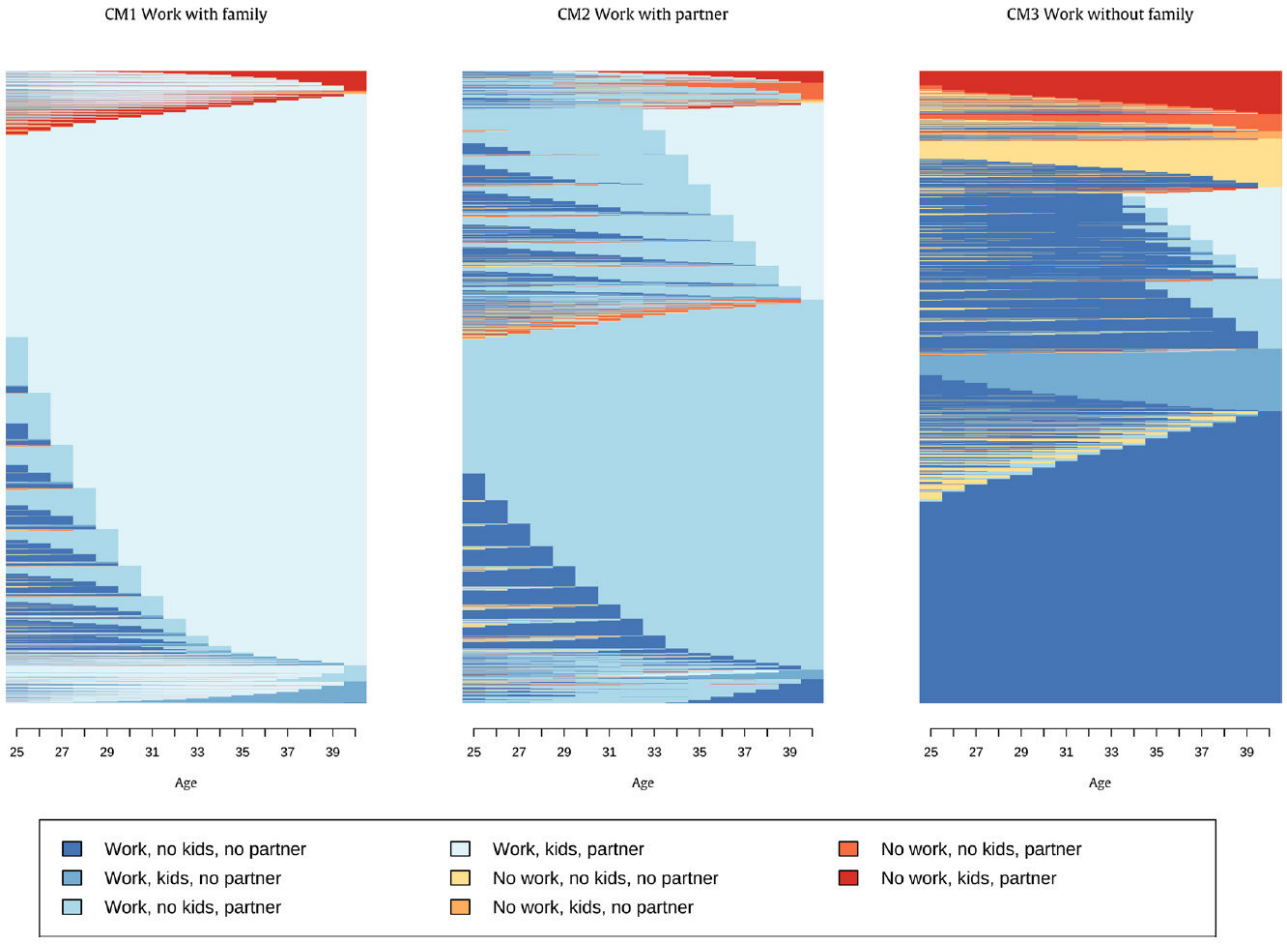
Indexplots by work-family cluster for men (n = 18,816).

**Fig. 2.**
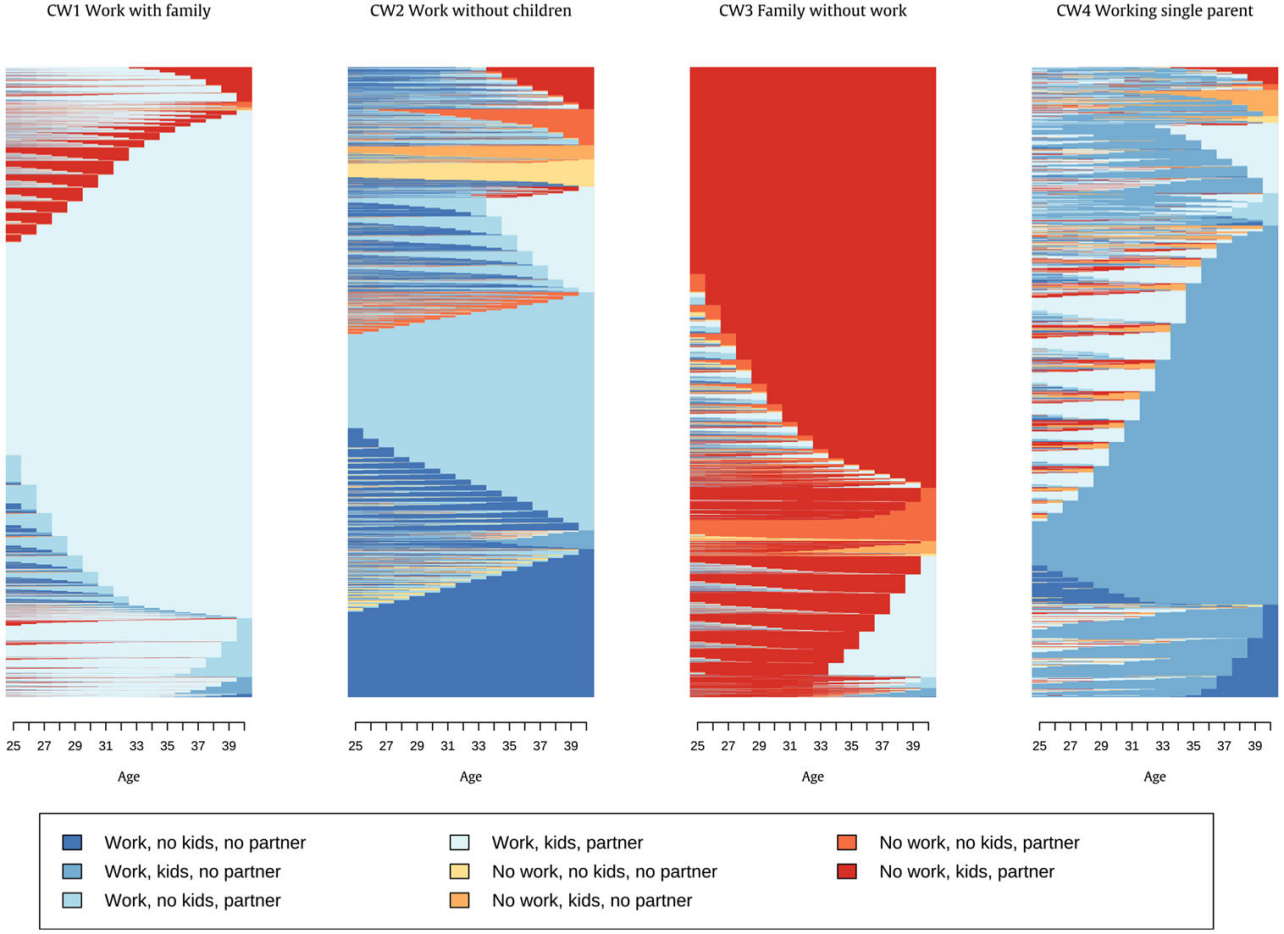
Indexplots by work-family cluster for women (n = 24,686).

The main patterns of life course social roles differ somewhat between men and women. As indicated in the Methods section, the sequence and cluster analysis point to a three-cluster solution for men and a four-cluster solution for women. Table 2 shows the distribution across work-family clusters as well as the dominant states and mean number of state changes within each cluster. Figs. 1 and 2 show indexplots for each identified cluster for men and women. Additional Table A3 in the Appendix shows the complete distribution of each respondent’s dominant state across all clusters.

For men, we found that the largest group of respondents combined stable employment with stable partnership and children (Cluster CM1 – “Work with family”) and a second group of men combined stable work with partnership but no children or children in their late 30s (Cluster CM2 – “Work with partner”). The third group of men had work but neither partner nor children for most of early adulthood (Cluster CM3 – “Work without family). The indexplots show that some men in this third cluster CM3 have some episodes of non-working (with or without family) but these states are very infrequent and do not last very long (also see Appendix A3).

For women, we found a similar largest group combining stable employment with stable partnership and children (Cluster CW1 – “Work with family”). The second group had work with or without a partner and no children (Cluster CW2 – “Work without children”), the third group had a stable partnership and children but no paid work (Cluster CW3 – “Family without work”) and a final group of women combined work and children but had no long-term partnership (Cluster CW4 – “Working single parent”).

Overall, the mean number of state changes ranged between 2 and 3 for both men and women and across all clusters, indicating that the constellations of combined social roles in early adulthood are fairly stable.

### 3.3. Multivariate analyses

Table 3 and 4 present multilevel regression coefficients of work-family clusters for depression in men and women respectively. The tables consist of three models for men and women. The first model contains all Level 1 variables. Model 2 additionally includes our Level 2 variable labour market context (female employment rate). Model 3 contains interaction terms between work-family clusters and the Level 2 variable.

**Fig. 3.**
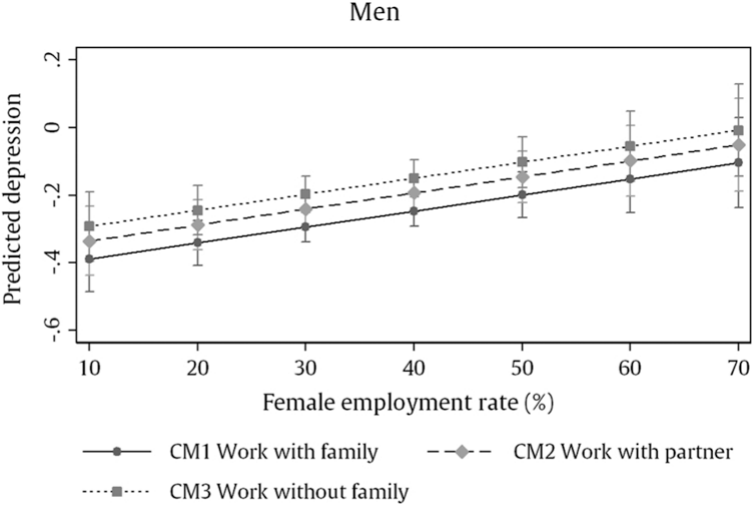
Predicted values for men’s mean depressive symptoms (standardised) by work-family cluster and country female employment rate at time of labour market entry (n = 16,379).

Regarding depressive symptoms, men with work and no family (CM3) and men with work and partners but no children (CM2) have significantly more depressive symptoms compared to men who combine all three roles (CM1). Interaction analyses show that this relation between work-family cluster and later depression does not differ by labour market context (compare Model 2 and Model 3). A closer inspection shows that we find slightly higher depressive symptoms for men across all clusters in countries with higher female employment rates instead (see Fig. 3).

The results for women are comparable to those for men. We also find that women who combine employment, partnership and parenthood (CW1) between the age 25 and 40 have on average fewer depressive symptoms at older age than women who had either no children (CW2), no work (CW3) or no partner (CW4) for most of their early adulthood. Differences in depressive symptoms are most prominent between women in the “Work with family”-cluster (CW1) and working single mothers (CW4), while differences between the other clusters were nonsignificant (see Appendix A4).

**Fig. 4.**
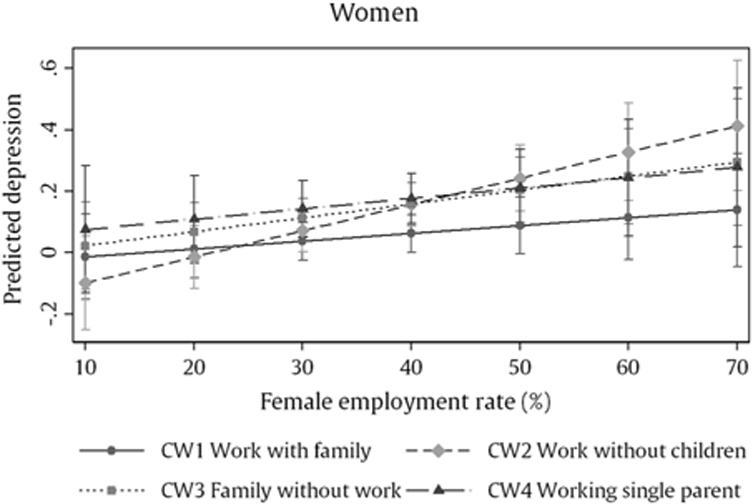
Predicted values for women’s mean depressive symptoms by work-family cluster and country female employment rate at time of labour market entry (n = 22,105).

But other than for men, interaction analyses reveal that the relationship between work-family clusters and depressive symptoms differs by labour market context at the time. In countries with higher female employment rates childless women (CW2) show higher depressive symptoms than women who combine all roles at the same time (CW1), while showing fewer depressive symptoms than other women in countries with lower female employment rates (see Fig. 4).

## 4. Discussion

Our findings show that work-family constellations through early adulthood are characterised by combinations of multiple roles for a large majority of men and women in Europe. While men are more likely than women to be working without family, women are more likely than men not to work while living with a partner and children during the “rush-hour” of life. For both genders, combining paid work, partnership and parenthood between the ages 25 and 40 is associated with lower depressive symptoms later in life. Additionally, we find that, for women, the relationship between social roles and mental health is moderated by the labour market context at the time.

### 4.1. Multiple roles in early adulthood and later mental health

The European men and women in our sample spent most years of their late 20s and 30s combining paid work with partnership and parenthood, confirming that the so-called “rush-hour” in early adulthood is indeed a time in the life course where role conflicts are particularly likely. Contrary to theories of role strain (Goode, 1960), our results show that men and women with multiple social roles have fewer depressive symptoms than other groups, suggesting that the accumulated resources associated with both stable work and family might outweigh the negative consequences of potential conflict between roles for mental health later in life.

These associations could partly be explained by an accumulation of financial resources, especially for those who combine stable work and partnership roles and benefit from dual incomes, but considering current socio-economic circumstances in our models did not alter the effects. Other benefits may be related to increased psychosocial resources, such as extended social networks at work and at home. Recent studies suggest that the mechanisms for the positive outcomes of combining multiple family and work roles include an increased feeling of autonomy, social support from co-workers and spill-over of positive affect and satisfaction from one social role to another (Beutell & Gopalan, 2019). In the family domain, one’s partner and children can also function as important sources of social support, especially at older age (Becker et al., 2019).

On the other hand, our results show that the simply comparing the number of social roles is not sufficient to explain the relationship between work-family trajectories and later mental health. Particularly for women, some constellations with two social roles, such as combining parenthood and employment without at partner, are more strongly associated with increased depressive symptoms than others, e.g. combining employment and partnership without children. This underlines the importance of including partnership histories when analysing work-family trajectories. While couples often divide household responsibilities and share accumulated financial and social resources, single mothers tend to experience more stress and work-family conflict resulting from competing demands (Cairney, Boyle, Offord, & Racine., 2003).

For men, trajectories that combine work and family roles are associated with fewer depressive symptoms than those without family. The internal variation of men’s work-family clusters makes it difficult to disentangle the effects of employment, partnership and parenthood in this study but the results indicate a “fatherhood premium” with benefits for later mental health.

One important point for discussion is the potential for reverse causation with regard to multiple social roles (Wethington, Moen, Glasgow, & Pillemer, 2000): Young adults with lower levels of well-being tend to be less likely to acquire paid work and form a stable family. These inequalities in early life could partly explain the relationship between multiple social roles and depressive symptoms in men and women. In our analysis, we considered some statistical controls of childhood circumstances but could not fully exclude individuals with early depression from the analysis (due to missing data for some countries). Previous studies have shown that health selection effects do not fully explain the relationship between social roles and well-being (McMunn, Bartley, Hardy et al., 2006).

### 4.2. Gender and the labour market context

Our results show that men who combine employment, partnership and parenthood in early adulthood have fewer depressive symptoms at older age than non-partnered and childless workers, regardless of the labour market context. Unexpectedly, we also find that men generally show higher depressive symptoms in countries with a history of higher female employment rates. This difference is somewhat driven by former communist countries from the Central East of Europe, where women made up a large proportion of the workforce and depression rates are high. Yet, removing these countries from the analysis did not fully remove the correlation between labour market and depression.

On the other hand, we find that women’s relationship between social roles and mental health does depend on the labour market context at the time. This is in line with the “principle of time and place” in life course theory (Elder et al., 2003). Women with work but no children in early adulthood show more depressive symptoms at older age in countries with high female employment rates while showing fewer depressive symptoms in countries with low female employment rates. It is possible that these differences reflect different social norms for combining work and family roles over the life course. In countries, where women are encouraged to combine employment, partnership and parenthood, female employment rates are higher and childless women might be stigmatized. Another possibility is that policies surrounding childcare provision in these countries are better and potential work-family conflicts for working mothers are reduced, hence, creating a more positive balance between the stress and resources of combining multiple roles. On the other hand, in countries with more familialistic policies, responsibility for childcare often lies within the household and often results in increased role strain for working mothers, which could outweigh other financial or psychosocial benefits of combining multiple roles. Recent findings by Grundy and colleagues (Grundy, Van Den Broek, & Keenan., 2019) show that the relationship between number of children and depressive symptoms differs between Eastern and Western European countries and indicate that support provided by adult children at older age might be particularly important in countries with greater economic stressors.

Interestingly, differences in mental health between working and non-working mothers are reduced after controlling for labour market context. Similarly, we find smaller differences between partnered and single mothers. Especially for women, lower rates of female employment can constitute a stronger social norm for staying-at-home. Familialistic policies can also increase the demands of combining children with paid work (Naldini, Pavolini, & Solera., 2016; Zagel & Van Winkle, 2020), and cancel out any benefits of role enhancement. Cross-country differences in the availability and acceptance of part-time work might also contribute to this. There are other possible confounders on country level that might help to explain the differences between labour market contexts, for example, general employment availability, marriage rates, income per capita or social security policies. However, given the few number of countries for a multilevel analysis we did not want to overburden our model (Bryan & Jenkins, 2016). Overall, the results suggest that the mental health benefits of combining work and family in early adulthood are stronger in countries with a labour market context that supports mothers in paid work. Future studies should explore these differences in more detail.

### 4.3. Strengths and limitations

The methods used in this study have several advantages that increase the salience of our findings, most notably the use of multiple sources of data and the large sample size. By using new harmonised life course data from SHARE and ELSA, we were able to compare work-family histories in a large sample across 22 countries, making our findings applicable to a much wider population, including the majority of countries in the European Union. Past studies combining data on employment and family histories spanning over more than a decade to analyse their relationship with mental health have been limited to smaller samples from single countries only (Benson et al., 2017; Engels et al., 2019; Freeh & Damaske, 2012; Lacey et al., 2016; McMunn, Bartley, Hardy et al., 2006; Stone et al., 2015).

Another advantage is the application of sequence analysis to map the course of the different social roles in early adulthood simultaneously. This allows us to describe work-family trajectories in detail including the number of years spent in each constellation and the number of changes in constellations. We could show that, for most European men and women, born between 1945 and 1975, simplified trajectories of combined work and family roles in early adulthood could be deducted and the number of changes in social roles was low.

The way we used sequence analysis to group work-family trajectories into clusters in this study also has some weaknesses that need to be addressed. Some work-family combinations are very rare and not represented well in any of the work-family clusters. This is particularly true for any non-working states in men, which are infrequent but mostly grouped into the “Work without family” cluster. Unemployment is known to be an important risk factor for depression at older age. We therefore performed some sensitivity analysis excluding all men who had any non-working states as their dominant states but the overall associations with depression did not change (see Appendix A5). Furthermore, partnership, parenthood and employment histories also not fully independent from each other, e.g. partnered individuals are more likely to have children in the coming years than single workers. Multi-channel sequence analysis (Gauthier, Widmer, Bucher, & Notredame, 2010) is one way to account for these interdependencies in future studies.

Other limitations relate to the structure of the available data in SHARE and ELSA. For example, the methodology relies on data from retrospective interviews with the participants with potential risks for recall bias or social desirability. On the other hand, studies on validity of this type of data revealed high accuracy of recalled information, in particular when respondents described their past socio-demographic situation (Berney & Blane, 1997) and employment histories (Bourbonnais, Meyer, & Theriault, 1988). There were also some respondents that had to be excluded because of incomplete employment, partnership or parenthood histories (missing state-information within sequences). Hereby, it is possible that we excluded incomplete histories with particular patterns (e.g. precarious employment histories). However, the value of imputing life course sequence data is still debated (Ritschard & Studer, 2018) which is why we decided against it.

Given that SHARE and ELSA are both surveys of ageing, the results are only applicable to European men and women born in first few decades after the Second World War. While the identified work-family trajectories are typical for these older cohorts, our findings cannot be extended to men and women who are currently in early adulthood. Research suggests that life courses are getting increasingly complex and individualised (McMunn et al., 2015). The number of single parents and individuals without family is increasing – the “golden age” of marriage and stable employment histories has largely ended. On top of that, social policies and labour market situation are also changing constantly. Future studies should study how the relationship of multiple social roles and mental health in younger cohorts compares to our findings. These studies should also consider using one typology to describe both men and women because work-family trajectories have become less gender-segregated.

Finally, some characteristics of work and family roles were not assessed, although research suggests that quality of the inhabited social roles may be more important than quantity for mental well-being. For example, we could not distinguish between part-time and full-time work. Particularly for mothers, part-time work is often a way to minimize potential conflict between paid employment and childcare (especially for those with a higher number of children). Therefore, further studies should also take into account working hours and distinguish between different reasons for non-employment, if possible. Similarly, role enhancement theory offers multiple pathways that lead to increased well-being but we could only examine a few of them. Here, sense of purpose and emotional support from any living children and grandchildren later in life could also factor in. Future research should also take into account information on occupational position and job type, as well as the quality of relationships and division of unpaid domestic work within families.

## 5. Conclusion

The present study investigates the relationship between multiple social roles and mental well-being from a life course perspective. Overall, our findings are more in line with the concept of role enhancement showing that, despite potential conflicts, combining work and family in early adulthood can be beneficial for men and women in the long term, while past episodes of non-employment and living without a partner or children were associated with higher depression scores at older age. The relationship between work-family constellations and mental health differs by gender and labour market context at the time, with stronger indications of role enhancement for women in countries with a higher female employment at the time of their labour market entry.

## Figures and Tables

**Table 1:** Sample description (n = 43,502).

	Total		Men		Women	
	Mean/ %	SD	Mean/ %	SD	Mean/ %	SD
**Age**	60.19	5.77	60.80	5.39	59.72	6.01
**Education** (1,162 missing values)						
Low	35.56		33.51		37.12	
Medium	41.12		42.22		40.28	
High	23.33		24.27		22.61	
***Cumulative durations (Years spent in…)***						
Work, no children, no partner	1.64	3.72	2.35	4.27	1.10	3.12
Work, children, no partner	0.58	2.22	0.46	2.00	0.68	2.37
Work, no children, partner	1.96	3.67	2.42	3.91	1.62	3.44
Work, children, partner	8.63	6.37	9.87	6.03	7.68	6.46
No work, no children, no partner	0.27	1.44	0.32	1.56	0.23	1.34
No work, children, no partner	0.13	0.99	0.04	0.51	0.19	1.24
No work, no children, partner	0.32	1.52	0.16	0.94	0.44	1.84
No work, children, partner	2.47	4.81	0.37	1.68	4.06	5.73
**Depressive symptoms (Euro-D/CES-D z-scores)**	−0.06	0.96	−0.26	0.85	0.08	1.01
(1,613 missing values)						
**Total**	43,502	100	18,816	100	24,686	100

Source: SHARE & ELSA.

**Table 2:** Work-family clusters for men and women, observations (n), percentage (Col. %), dominant states and state changes.

	n	Col. %	Cluster Name	Dominant state^a^	Mean number of state changes
**Cluster Men**					
*CM1*	13,814	73.42	Work with family	Work, children, partner (13.06)	2.19
*CM2*	2,388	12.69	Work with partner	Work, no children, partner (11.20)	2.49
*CM3*	2,614	13.89	Work without family	Work, no children, no partner (10.38)	2.08
Total	18,816	100.0			
	n	Col. %	Cluster Name	Dominant state^a^	Mean number of state changes
**Cluster Women**					
*CW1*	13,114	53.12	Work with family	Work, children, partner (13.22)	2.17
*CW2*	3,654	14.80	Work without children	Work, no children, partner (6.82)	2.37
*CW3*	6,617	26.80	Family without work	No work, children, partner (12.47)	2.21
*CW4*	1,301	5.27	Working single parent	Work, children, no partner (9.56)	3.05
Total	24,686	100.0			

Source: SHARE & ELSA.

a Cells show the most dominant state and the mean number of years spent in it between ages 25 and 40.

**Table 3:** Multilevel regression models of (standardised) depressive symptoms for men with unstandardized coefficients (b) and 95 % confidence intervals (CI 95 %) (n = 16,379).

	Model 1		Model 2		Model 3	
	b	CI 95 %	b	CI 95 %	b	CI 95 %
**Work-family cluster**						
CM1 Work with family	Ref.	Ref.	Ref.	Ref.	Ref.	Ref.
CM2 Work with partner	0.05**	0.02,0.09	0.05**	0.02,0.09	−0.01*	−0.14,0.12
CM3 Work without family	0.09**	0.06,0.13	0.09**	0.06,0.13	0.14	0.02,0.26
**Education**						
Low	Ref.	Ref.	Ref.	Ref.	Ref.	Ref.
Medium	−0.09**	−0.12,−0.06	−0.09**	−0.13,−0.06	−0.09**	−0.13,−0.06
High	−0.12**	−0.16,−0.08	−0.12**	−0.16,−0.08	−0.12**	−0.16,−0.08
**Childhood conditions**						
People per room	0.02**	0.01,0.03	0.02**	0.01,0.03	0.02**	0.01,0.03
Childhood Hospitalisation	0.16**	0.11,0.21	0.16**	0.11,0.21	0.16**	0.11,0.21
**Economic conditions**						
Income	−0.01**	−0.02,−0.01	−0.01**	−0.02,−0.01	−0.02**	−0.02,−0.01
Wealth	−0.03**	−0.03,−0.03	−0.03**	−0.03,−0.03	−0.03**	−0.03,−0.02
**Labour market context**						
Female Employment Rate (FER)			0.00**	0.00,0.01	0.00*	0.00,0.01
FER*CM1 Work with family					Ref.	Ref.
FER*CM2 Work with partner					0.00	−0.00,0.01
FER*CM3 Work without family					0.00	0.00,0.00
**Model**						
Log likelihood	−20,004		−20,001		−20,000	
AIC	40,035		40,030		40,032	
BIC	40,135		40,138		40,156	
Variance Level 1	0.670		0.670		0.670	
Variance Level 2	0.016		0.014		0.014	
ICC	0.024		0.020		0.020	
N Level 1	16,379		16,379		16,379	
N Level 2	44		44		44	

Source: SHARE & ELSA. Note:

** =p<0.01

* =p<0.05.

Note: All models are adjusted for age and age^2^.

**Table 4:** Multilevel regression models of (standardised) depressive symptoms for women with unstandardized coefficients (b) and 95 % confidence intervals (CI 95 %) (n = 22,105).

	Model 1		Model 2		Model 3	
	b	CI 95 %	b	CI 95 %	b	CI 95 %
**Work-family cluster**						
CW1 Work with family	Ref.	Ref.	Ref.	Ref.	Ref.	Ref.
CW2 Work without children	0.06**	0.02,0.10	0.06**	0.02,0.10	−0.15*	−0.28,−0.02
CW3 Family without work	0.08**	0.05,0.12	0.08**	0.05,0.12	0.02	−0.09,0.12
CW4 Working single parent	0.11**	0.05,0.17	0.11**	0.05,0.17	0.07	−0.15,0.30
**Education**						
Low	Ref.	Ref.	Ref.	Ref.	Ref.	Ref.
Medium	−0.13**	−0.17,−0.10	−0.14**	−0.17,−0.10	−0.14**	−0.17,−0.10
High	−0.21**	−0.25,−0.17	−0.22**	−0.26,−0.18	−0.22**	−0.26,−0.18
**Childhood conditions**						
People per room	0.05**	0.04,0.06	0.05**	0.04,0.06	0.05**	0.04,0.06
Childhood Hospitalisation	0.16**	0.11,0.21	0.16**	0.11,0.21	0.16**	0.11,0.21
**Economic conditions**						
Income	−0.01**	−0.02,−0.01	−0.01**	−0.02,−0.01	−0.01**	−0.02,−0.01
Wealth	−0.04**	−0.04,−0.03	−0.04**	−0.04,−0.03	−0.04**	−0.04,−0.03
**Labour market context**						
Female Employment Rate (FER)			0.00	−0.00,0.01	0.00	−0.00,0.01
FER*CW1 Work with family					Ref.	Ref.
FER*CW2 Work without children					0.01**	0.00,0.01
FER*CW3 Family without work					0.00	−0.00,0.01
FER*CW4 Working single parent					0.00	−0.00,0.01
**Model**						
Log likelihood	−30,674		−30,672		−30,666	
AIC	61,375		61,375		61,370	
BIC	61,487		61,494		61,514	
Variance Level 1	0.935		0.935		0.934	
Variance Level 2	0.028		0.026		0.026	
ICC	0.029		0.027		0.027	
N Level 1	22,105		22,105		22,105	
N Level 2	44		44		44	

Source: SHARE & ELSA. Note:

** =p<0.01

* =p<0.05

Note: All models are adjusted for age and age^2^.

**Appendix A4:**
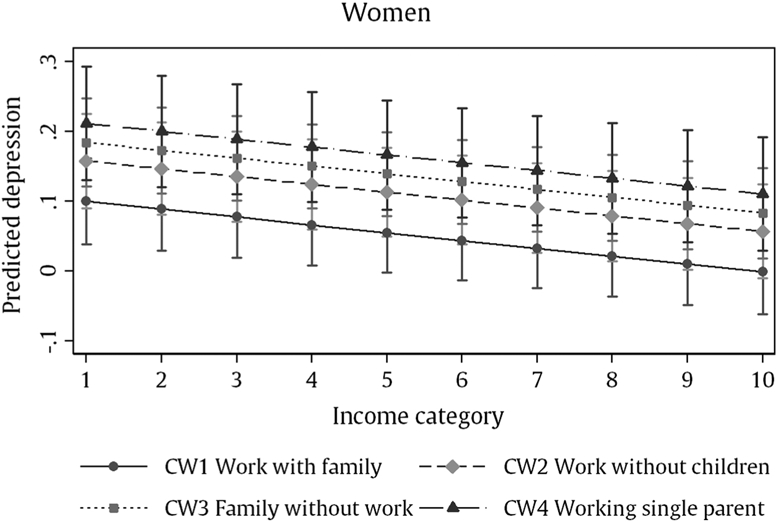
Predicted values for women’s mean depressive symptoms (xy-standardised) by work-family cluster and income category (n = 22,105), Model 1

**Appendix A5:**
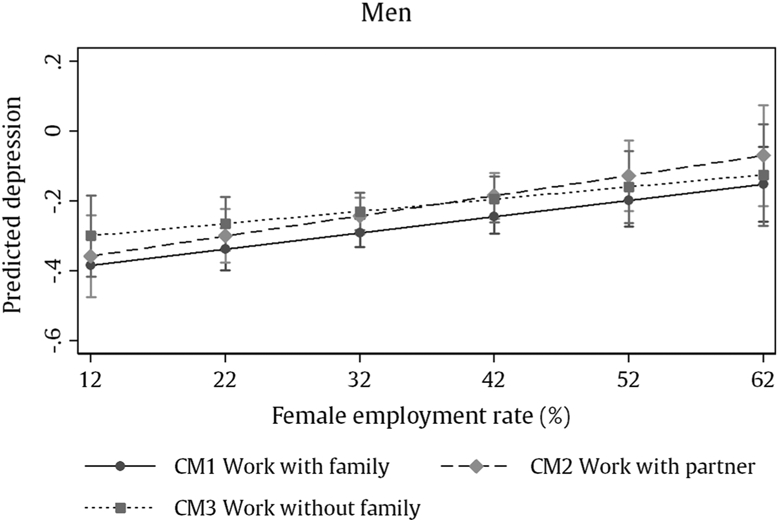
Predicted values for men’s mean depressive symptoms (xy-standardised) by work-family cluster and country female employment rate without “non-working” dominant states (n = 15,811)

## Appendix

**Appendix A1:** National female employment rates in the 1970s and 1980s by country

	70s Total %	80s Total %
Sweden	29.9	66.9
Denmark	36.1	54.4
England	32.9	42.9
Ireland	19.4	29.5
Austria	30.3	34.9
West Germany	30.4	49.3
East Germany (former GDR)	41.3	36.8
Switzerland	32.1	34.4
Netherlands	19.1	31.9
Belgium	24.4	33.6
Luxembourg	19.5	31.2
France	30.2	43.3
Spain	13.4	27.3
Portugal	19.0	36.4
Italy	19.3	29.0
Greece	20.4	28.7
Estonia^2^	46.6	46.6
Poland	46.4	57.0
Czech Republic	42.3	48.7
Hungary	38.6	42.9
Slovenia	30.7	43.6
Croatia ^2^esti mates	30.7	37.4

Source: SHARE & ELSA.

**Appendix A2:** Cluster solutions of work-family clusters

Number of clusters	Mean within distances	Mean between distances	WB-Ratio	PBC	ASW	Obs. in smallest cluster
Men						
2	8.61	12.90	0.67	0.58	0.32	1,352
3	7.89	12.66	0.62	0.64	0.32	680
4	7.53	11.78	0.64	0.50	0.19	554
Women						
2	9.74	12.11	0.80	0.28	0.19	2,122
3	8.43	12.44	0.68	0.53	0.26	1,412
4	7.65	12.40	0.62	0.60	0.29	744
5	7.35	11.97	0.61	0.51	0.21	723

Source: SHARE & ELSA. Note: PBC: WB-Ratio: Within/between cluster distance ratio; PBC: Point Biserial Correlation; ASW: Average Silhouette Width.

**Appendix A3:** Distribution of dominant States, observations (n) per work-family cluster, percentage (Col. %)

Dominant state	CM1 Work with family	CM2 Work with partner	CM3 Work without family	CW1 Work with family	CW2 Work without children	CW3 Family without work	CW4 Working single parent
**Work, children, partner**	13,275* (96.1)	150 (6.3)	6 (0.2)	12,916* (98.5)	94 (2.6)	43 (0.7)	230 (17.7)
**Work, no children, no partner**	131 (1.0)	126 (5.3)	1,942* (74.3)	1 (0.0)	1,397 (38.2)	10 (0.2)	4 (0.3)
**Work, children, no partner**	221 (16)	24 (1.0)	236 (9.0)	4 (0.0)	11 (0.3)	5 (0.1)	988* (75.9)
**Work, no children, partner**	5 (0.1)	2,016* (84.4)	6 (0.2)	16 (0.1)	1,720* (47.1)	17 (0.2)	1 (0.1)
**No work, children, partner**	148 (1.1)	26 (1.0)	143 (5.6)	120 (0.9)	30 (0.8)	6,189* (93.5)	15 (12)
**No work, no children, no partner**	11 (0.1)	14 (0.6)	210 (8.0)	11 (0.1)	149 (4.1)	53 (0.8)	2 (0.2)
**No work, children, no partner**	15 (0.1)	2 (0.1)	18 (0.7)	39 (0.3)	85 (2.3)	57 (0.9)	59 (4.5)
**No work, no children, partner**	8 (0.1)	30 (13)	53 (2.0)	7 (0.1)	168 (4.6)	246 (3.7)	2 (0.2)

* dominant state of the cluster.

**Appendix A6:** Distribution of gender-specific work-family clusters across countries

	Men			Women			
	CM1 %	CM 2 %	CM3 %	CW1 %	CW2 %	CW3 %	CW4 %
Sweden	71.0	16.0	13.0	64.7	16.6	10.1	8.7
Denmark	72.0	15.9	12.2	69.0	14.7	10.7	5.6
England	67.0	22.2	10.8	41.7	19.2	34.6	4.6
Ireland	72.9	6.8	20.3	29.7	17.0	51.8	1.6
Austria	72.1	15.1	12.9	41.3	18.3	33.1	7.4
West Germany	66.2	17.9	15.8	42.5	20.3	32.5	4.8
East Germany	79.3	7.2	13.5	80.2	6.3	6.3	7.3
Switzerland	62.2	21.3	16.6	29.7	27.9	38.2	4.2
Netherlands	67.7	18.0	14.3	22.7	21.1	52.4	3.8
Belgium	70.6	15.3	14.2	50.8	18.9	24.3	6.1
Luxembourg	67.2	17.7	15.1	33.8	17.7	43.7	4.8
France	75.4	10.5	14.1	54.4	15.4	24.7	5.5
Spain	71.1	12.2	16.7	37.7	15.2	44.4	2.3
Portugal	76.7	11.0	12.3	45.2	12.9	37.1	4.8
Italy	71.7	10.2	18.1	36.9	16.4	44.7	2.0
Greece	69.1	11.3	19.7	30.1	14.7	53.6	1.7
Estonia	81.8	7.9	10.3	74.2	9.4	5.3	11.1
Poland	83.8	4.8	11.4	67.0	7.4	23.1	2.6
Czech Republic	84.9	6.1	9.0	80.3	7.3	5.0	7.5
Hungary	82.8	7.2	10.0	78.4	8.6	8.4	4.6
Slovenia	78.9	6.7	14.5	74.2	7.9	12.1	5.9
Croatia	80.0	6.3	13.8	59.9	8.9	27.7	3.5

Source: SHARE & ELSA. Note: Men’s Clusters: CM1 = Work with family, CM2 = Work with partner, CM3 = Work without family; Women’s Clusters: CW1 = Work with family, CW2 = Work without children, CW3 = Family without work, CW4 = Working single parent.
